# Relationship Between Cell Compatibility and Elastic Modulus of Silicone Rubber/Organoclay Nanobiocomposites

**Published:** 2012-05-28

**Authors:** Motahare Sadat Hosseini, Mohammad Tazzoli-Shadpour, Issa Amjadi, Nooshin Haghighipour, Mohammad Ali Shokrgozar, Mehri Ghafourian Boroujerdnia

**Affiliations:** 1 Biomedical Group, Faculty of Biomedical Engineering (Center of Excellence), Amirkabir University of Technology, Tehran, IR Iran; 2 Polymer Group, Faculty of Polymer Engineering and Color Technology (Center of Excellence), Amirkabir University of Technology, Tehran, IR Iran; 3 National Cell Bank of Iran, Pasteur Institute of Iran, Tehran, IR Iran; 4 Nanotechnology Research Center, Ahvaz Jundishapur University of Medical Sciences, Ahvaz, IR Iran; 5 Immunology Department, Medical College, Ahvaz Jundishapur University of Medical Science, Ahvaz, IR Iran

**Keywords:** Nanocomposite, Elastic Modulus, Cytotoxicity Tests

## Abstract

**Background:**

Substrates in medical science are hydrophilic polymers undergoing volume expansion when exposed to culture medium that influenced on cell attachment. Although crosslinking by chemical agents could reduce water uptake and promote mechanical properties, these networks would release crosslinking agents. In order to overcome this weakness, silicone rubber is used and reinforced by nanoclay.

**Objectives:**

Attempts have been made to prepare nanocomposites based on medical grade HTV silicone rubber (SR) and organo-modified montmorillonite (OMMT) nanoclay with varying amounts of clay compositions.

**Materials and Methods:**

Incorporation of nanocilica platelets into SR matrix was carried out via melt mixing process taking advantage of a Brabender internal mixer. The tensile elastic modulus of nanocomposites was measured by performing tensile tests on the samples. Produced polydimetylsiloxane (PDMS) composites with different flexibilities and crosslink densities were employed as substrates to investigate biocompatibility, cell compaction, and differential behaviors.

**Results:**

The results presented here revealed successful nanocomposite formation with SR and OMMT, resulting in strong PDMS-based materials. The results showed that viability, proliferation, and spreading of cells are governed by elastic modulus and stiffness of samples. Furthermore, adipose derived stem cells (ADSCs) cultured on PDMS and corresponding nanocomposites could retain differentiation potential of osteocytes in response to soluble factors, indicating that inclusion of OMMT would not prevent osteogenic differentiation. Moreover, better spread out and proliferation of cells was observed in nanocomposite samples.

**Conclusions:**

Considering cell behavior and mechanical properties of nanobiocomposites it could be concluded that silicone rubber substrate filled by nanoclay are a good choice for further experiments in tissue engineering and medical regeneration due to its cell compatibility and differentiation capacity.

## 1. Background

Cells within living tissues are sensitive to biochemical signals as well as to mechanical factors in their environments (1). Physico-chemical properties of extracellular matrix (ECM) (2) and selective junctions of transmembrane proteins such as integrins (3) enable cells to sense and respond to stimuli in their environments and initiate a limited repertoire of cellular signals.

The sensitivity of cells arises from mechanosensitive nature of cell adhesion (4). Cell-matrix adhesion includes dense networks of proteins (5) in which chemical signaling plays key roles (6). As an example, a substrate displays different behaviors in response to cell aggregation according to its physical and mechanical properties (7). In other words, if a substrate is stiff and non-elastic, focal adhesions serve as structural links between ECM and actin cytoskeleton (8). Focal adhesion is a stable contact which mediates cell adhesion to the substrate (9). In contrast, soft and elastic substrates provide transient cell anchoring to the matrix (10). On the other hand, cell-ECM complexes control three-dimensional organization of cells in tissues as well as cell growth, movement, shape, gene expression, and embryonic development.

Recent studies have reported that regulation of substrate elasticity in two-dimensional cell cultures affects the differentiation and behavior specifications of cells (11). Mechanical properties of cell substrate influence on cell functionality. Cytoskeletal arrangement and orientation are highly dependent upon mechanical and structural properties of the matrix such as elastic modulus, Poisson’s ratio, and roughness (12, 13). When interacting with the substrate, cellular responses including relaxation time and adaptation through alteration in fibrous structures are defined by local matrix deformability (14, 15). Adjustment of cell cytoskeleton to mechanical properties of the substrate roots in polymerization and depolimerization of actin fibers (16), which acts via focal adhesion proteins at cell-substrate interface (14). Hydrophilic polymers are the most studied substrates to investigate effects of physical or chemical properties of substrates on cell behaviors (8, 17). These substrates undergo volume expansion when exposed to culture medium that influenced on cell attachment. Although chemical crosslinking by low molecular weight agents could reduce water uptake and promote mechanical properties, these three dimensional cross-linked polymer networks would degrade over time and release crosslinking agents. Therefore, a hydrophobic polymer (HTV silicone rubber) is employed which is one of the polymers widely used in biomedical applications where, however, it exhibits poor mechanical properties. In order to overcome this weakness, it is reinforced by particle or layered fillers. Nanofillers notably affect the properties of elastomer despite their low volume fraction (18).

## 2. Objectives

Hence in this study, we synthesized OMMT/SR nanocomposites in order to create a substrate with different stiffness and elasticity. Then the responses of endothelial cells and ADSCs to altered mechanical properties were analyzed.

## 3. Materials and Methods

### 3.1. Materials and Preparation of Composites

Medical SR was vulcanized at high temperatures and clay nanoparticles, Closite 15A, were supplied by Southern Clay Products, USA. Dicumyl peroxide was employed in preparation and cure of the samples. Initially, nanocomposites were prepared through melt mixing method. The rubbery compound was mixed in a Brabender mixer with OMMT mass ratios of 1%, 2%, and 3% at 60 rpm and 60°C for 20 minutes [19]. The samples were vulcanized at 160°C by compression molding at optimum cure time (t_95_) obtained from curing curves in a very low thickness.

### 3.2. Isolation of Mesenchymal Stem Cells

Initially, omental fat was rinsed in PBS 5% containing penicillin/ Streptomycin. It was cut by a scalpel and pipetted several times. The floating adipose tissue was put in a dish containing collagenase type I (concentration: 0.075 %) and penicillin/ Streptomycin (concentration: 2%), and stirred continuously in an incubator for 30 minutes. Collagenase was neutralized by 5 ml of DMEM-20% FBS. The samples were extensively pipetted to separate connective tissue parts. Obtained samples, then, were centrifuged twice at 2000 rpm for 5 minutes. The cells were suspended in DMEM-10% FBS, transferred into 12-well plates and placed in an incubator for 72 hours to adhere to the plate (19). At 85% confluency , the cells were harvested for differentiation aimed towards osteogenic lineages in response to both elastic modulus and growth factor.

### 3.3. Measurement of Mechanical Properties

Mechanical properties, including tensile strength and elastic Moduli at 100 and 300 percent, were performed using at least three dumbbell shaped samples and a tensile testing device (Cardano al Campo (VA)-Italy) according to ASTM D412 (20).

### 3.4. Cytotoxicity Assays and Cell Morphology

To evaluate biocompatibility and cytotoxicity, Human Umbilical Vein Endothelial Cells (HUVECs) were provided by the originator (Dr. M.A. Shokrgozar, National Cell Bank, Pasteur Institute of Iran), and cultured in DMEM + Ham’s F12 (Gibco, USA) containing 10% FBS (Seromed, Germany) in a humidified 5% CO_2_ incubator. To study cell morphology on membranes, the samples were placed firstly in a culture plate and 5 x 10^3^ HUVECs with culture medium were added to each well. Subsequently, cells-included culture plate was incubated at 37°C in a CO_2_ incubator to adhere cells to the membranes after whichseveral images were captured via inverted microscope (Zeiss, Germany) overnight (21). Cell proliferation was analyzed taking advantage of MTT (3-[4, 5-dimethyltriazol-2-y1]-2, 5-diphenyl tretrazolium bromide) (). In brief, 5 x 10^3^ cells were added to a 96-well plate including SR composites and were incubated at 37°C in the same atmosphere with 5% CO_2_ for 24 hours. After cell adhesion occurred, the culture medium related to each well was replaced by 100 µl of MTT with the concentration of 0.5 mg/ml, and the plates were incubated at 37°C for 4 hours. Finally, dimethyl sulfoxide was added to each well and cell growth was analyzed at 570 nm (22).

### 3.5. Osteogenesis Potential Assay

Confluent ADSCs (passage number = 2) were detached and counted; then 5 × 10^3^ cells were put in the vicinity of the substrates as well as in a differential medium containing DMEM Ham’s F12,10% FBS, 10 mM ß-glycerophosphate, 50 µg/ml sodium ascorbate 2-phosphate, and 100 U penicillin/100 µg streptomycin/0.25 µg fungizone. The cells were incubated for up to 21 days, and osteogenic medium was replaced every 3 days. Afterwards, the cells were rinsed in 0.9 % sodium chloride and fixed by 70% alcohol. Alizarin red staining was performed to assess functional capacity of nanobiocomposites in the presence of growth factor (23).

### 3.6. Statistics

The data were analyzed by using the *t*-test. Data was presented as mean values ±S.D. and *P* < 0.05 was considered statistically significant.

## 4. Results

### 4.1. Biological Properties

**Figure 1:**
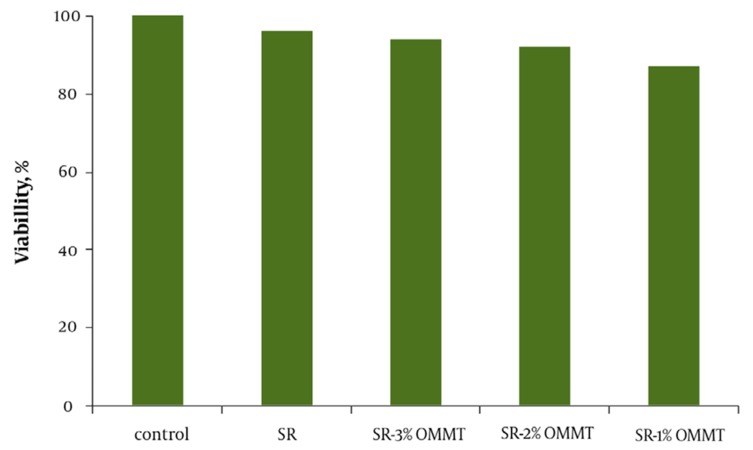
MTT Test Results for Different Nanobiocomposites and Control Sample Abbrivations: SR: Silicone Rubber; OMMT: Organo-Modified Montmorillonite Nanoclay

**Figure 2:**
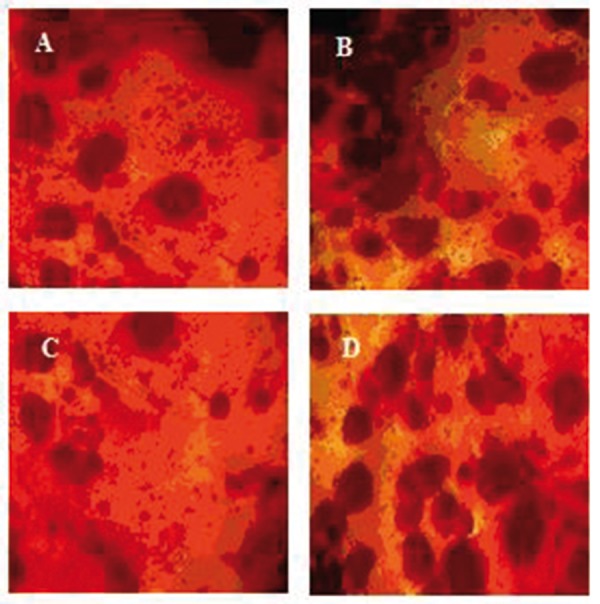
Comparison of Cell Morphology on the Nanobiocomposites and Neat Polymer Samples; (a) SR, (b) SR-1% OMMT, (c) SR-2% OMMT, and (d) SR-3% OMMT, (×400)

There is a hypothesis expressing that mechanical properties of a substrate on which cells grow governs their behavior (24). To study this theory, we prepared new collagen coated SR membranes of variable stiffness by adding small ratios of OMMT. The young’s modulus of composites ranged over 1 mega Pascal (MPa). MTT results indicate high degree of proliferation. All the samples have proven to be nontoxic and biocompatible with the viability greater than 90% (Figure 1). Moreover, the cells cultured on OMMT/SR nanocomposites displayed appropriate growth and spread morphology compared to neat polymer and were intensified more and more by increasing OMMT content (Figure 2). Cell morphology and contact area of HUVECs altered dramatically with the changing substrates’ young’s modulus. The cells adhered to the stiffest nanobiocomposites exhibited typically better spreading, whereas those placed on softer surfaces of nanobiocomposites indicated decreasing contact area. It could be deduced from the above results that the behavior of HUVECs is very sensitive to mechanical properties of the membranes attached on. It was also reasoned that not only the elastic modulus dictated by loading nanofillers could handle cell morphology, but also it might be able to regulate cell proliferation. In the short term cultures, the cells on rigid compared to soft membranes became confluent more quickly. It could be attributed to how cells could spread on the substrates and what contact area they had on them which were both directly referred to different mechanical properties of membranes.

**Figure 3:**
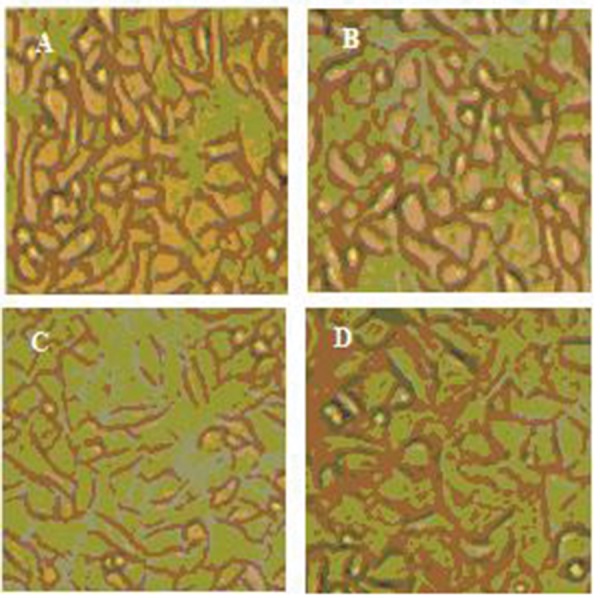
Alizarin Red Staining of ASC Cells Cultured in Bone Culture Medium. (A) SR, (B) SR-1% OMMT, (C) SR-2% OMMT, (D) SR-3% OMMT.

In tissue engineering, the cell fate and maintenance of stem cells to different lineage such as osteogenic would be manipulated by many factors including chemical stimulations of osteogenesis conducted by various proteins (25) and physiological simulations such as presence of scaffolds that transfer mechanical signals of the environment (26). Considering this point, some functional nanobiocomposites that meet above conditions were designed for osteogenic differentiation. Since the membranes’ young’s modulus is in the range of the bone modulus about 10 MPa (24), they would be able to guide osteogenic fate of ADSCs. In order to analyze the substrates’ effect on differentiation potential of ADSCs, alizarin red staining was performed (Figure 3). The results suggest that ADSCs could differentiate into an osteogenic phenotype under a defined condition after 21 days. We further will demonstrate how the functional properties of these nanobiocomposites on which ADSCs could adhere to would vary with culture conditions.

### 4.2. Mechanical Properties

Table 1 summarizes overall tensile testing results. It is observed that by adding OMMT particles, tensile strength increases (*P* < 0.05). Moreover, addition of OMMT particles leads to statistically significant stiffening of the substrates (*P* < 0.05). This trend of increase in elastic modulus is consistent with tensile tests when performed in both 100% and 300% elongations.

**Table 1:** Mechanical Properties of OMMT/SR Nanobiocomposites With Different Loading Percentages

	Properties			
Nanobiocomposites	Tensile strength (MPa a)	Tensile modulus (MPa a)	modulus at 100% Elongation	modulus at 300% Elongation
SR a	16.65 ± 0.43	10.02 ± 0.46	10.33 ± 0.29	16.24 ± 0.02
SR/OMMT a 1%	17.12 ± 0.98	10.21 ± 0.05	10.46 ± 0.12	16.48 ± 0.25
SR/OMMT a 2%	17.34 ± 0.35	10.38 ± 0.88	10.55 ± 0.99	16.77 ± 0.39
SR/OMMT a 3%	18.67 ± 0.59	10.96 ± 0.14	11.10 ± 0.81	17.24 ± 0.52

^a^Abbreviations: MPa: Mega Pascal; SR: Silicone Rubber; OMMT: Organo-Modified Montmorillonite Nanoclay

## 5. Discussion

To study effects of substrate elasticity on cell behavior, nanocomposite substrates were used by dispersion of differing mass ratio of nanoparticles of OMMTs in silicone rubber. Previous studies have shown that composites based on HTV silicone rubber polymer and silicate platelets produced transparent nanocomposites with high surface energy originated from filler tactoids with thickness of about 10-15 nm in reference polymer matrix (27, 28). Well dispersed nanoclay platelets observed in the AFM micrographs at scale of 750 nm induced transparency. In other words, the size of scattering center (layered silicate) is below the size of wavelength of visible light.

Results indicated significant influence in polymer matrix on mechanical properties of the substrate. Addition of OMMT resulted in stronger and stiffer matrix. Decrease of the crosslinking density which was evaluated from cure curves cited that nanofiller acts as an inhibitor. In other words, addition of nanoparticles into a polymer matrix would prevent chemical crosslinking and causes physical integrity (25) with a strong interface between nanofiller and silicone rubber (26). Such integrity is achieved by uniform distribution of particles into the nanocomposite structure.

In addition to enhancement of mechanical properties, cytotoxicity and biocompatibility analyses revealed improved cell behavior on the modified substrates. Addition of nanoparticles did not result in toxic effects and the resultant substrates found to be biocompatible. Cell growth and alignment was smoothly enhanced in nanocomposite substrates. It has been shown that inorganic nanofillers have higher surface energy than polymer matrix (19). Silicone rubber is hydrophobic due to the presence of methyl groups, hence PDMS is considered as a low surface energy polymer (21). Increasing in OMMT leads to the rise of free surface energy by mechanism of enhancing surface roughness (22). Rapid cell growth and proliferation are obtained on the surfaces with higher energy (24), as described by cell cover in this study. By increased surface energy of the substrate, the level of energy differences between cell membrane and matrix surface is elevated and therefore strong contacts are generated which results in desirable cell proliferation and signaling.

Mechanical properties of substrate influences cell functionality. Cytoskeletal arrangement and orientation is highly dependent on mechanical and structural properties of the matrix such as elastic modulus, Poisson’s ratio, and roughness (25, 26). When interact with substrate, cellular responses including relaxation time and adaptation by alteration in fibrous structure are defined by local matrix deformability (27, 28). The adjustment of cell cytoskeleton to mechanical properties of the substrate roots in polymerization and depolimerization of actin fibers (29) which act via focal adhesion proteins at cell-substrate interface (26).

In this work, we applied a comparatively easy method to fabricate a new type of substrate on which a cell could attach, proliferate and differentiate. It is also interesting to reveal that the mechanical properties have a very strong effect on cell morphology. Therefore, by controlling the elastic modulus of the composites, we can adjust cell compatibility, which may have the key role for applications in tissue engineering and regenerative medicine. This kind of alignment of the HUVECs on the rubber membrane could be of advantage for a more effective presence of OMMT. However, loading nanoparticles also enhances tensile strength and young’s modulus as evinced by the tensile experiments. Moreover, the results demonstrated that application of nanofiller into the polymer matrix could be a way to come by better proliferation and adhesion of cell lines. The elastic moduli of corresponding nanobiocomposites could be improved by increasing in the amount of OMMT, as their tensile strength could be done. In other words, adding certain ratios of nanoparticles resulted in substantial increase in the desirable mechanical properties. These results originate in good dispersion of the clay as well as strong interfacial interaction between polymer matrix and filler, which were best reflected in tensile parameters, because the strength values were remarkably promoted.

## References

[1] Gentile F, Tirinato L, Battista E, Causa F, Liberale C, di Fabrizio EM (2010). Cells preferentially grow on rough substrates.. Biomaterials..

[2] Ulrich TA, de Juan Pardo EM, Kumar S (2009). The mechanical rigidity of the extracellular matrix regulates the structure, motility, and proliferation of glioma cells.. Cancer Res..

[3] Cavalcanti-Adam EA, Volberg T, Micoulet A, Kessler H, Geiger B, Spatz JP (2007). Cell spreading and focal adhesion dynamics are regulated by spacing of integrin ligands.. Biophys J..

[4] Nicolas A, Safran SA (2006). Limitation of cell adhesion by the elasticity of the extracellular matrix.. Biophys J..

[5] Zamir E, Geiger B (2001). Molecular complexity and dynamics of cell-matrix adhesions.. J Cell Sci..

[6] Borman S (2011). Controlling cell spacing.. Chemical & Engineering News..

[7] Wang HB, Dembo M, Wang YL (2000). Substrate flexibility regulates growth and apoptosis of normal but not transformed cells.. Am J Physiol..

[8] Guo WH, Frey MT, Burnham NA, Wang YL (2006). Substrate rigidity regulates the formation and maintenance of tissues.. Biophys J..

[9] Iwanaga Y, Braun D, Fromherz P (2001). No correlation of focal contacts and close adhesion by comparing GFP-vinculin and fluorescence interference of DiI.. Eur Biophys J..

[10] Qian J, Gao H (2010). Soft matrices suppress cooperative behaviors among receptor-ligand bonds in cell adhesion.. PLoS One..

[11] Kidoaki S (2010). Mechanics in Cell Adhesion and Motility on the Elastic Substrates.. J Biommat Sci Eng..

[12] De R, Zemel A, Safran SA (2007). Dynamics of cell orientation.. Nat Phys..

[13] Wei Z, Deshpande VS, McMeeking RM, Evans AG (2008). Analysis and interpretation of stress fiber organization in cells subject to cyclic stretch.. J Biomech Eng..

[14] De R, Safran SA (2008). Dynamical theory of active cellular response to external stress.. Physical Review E..

[15] Hsu HJ, Lee CF, Kaunas R (2009). A dynamic stochastic model of frequency-dependent stress fiber alignment induced by cyclic stretch.. PLoS One..

[16] Nekouzadeh A, Pryse KM, Elson EL, Genin GM (2008). Stretch-activated force shedding, force recovery, and cytoskeletal remodeling in contractile fibroblasts.. J Biomech..

[17] Lo CM, Wang HB, Dembo M, Wang Y (2000). Cell movement is guided by the rigidity of the substrate.. Biophys J..

[18] George JJ, Bhowmick AK (2009). Influence of Matrix Polarity on the Properties of Ethylene Vinyl Acetate-Carbon Nanofiller Nanocomposites.. Nanoscale Res Lett..

[19] Momen G, Farzaneh M (2011). Survey of micro/nano filler use to improve silicone rubber for outdoor insulators. Rev Adv Mater Sci..

[20] Ma J, Yu ZZ, Kuan HC, Dasari A, Mai YW (2005). A new strategy to exfoliate silicone rubber/clay nanocomposites.. Macromol Rapid Commun..

[21] Haghighipour N, Tafazzoli‐Shadpour M, Shokrgozar MA, Amini S (2010). Effects of cyclic stretch waveform on endothelial cell morphology using fractal analysis.. Artif Organs..

[22] Huag HS, Chou SH, Don TM, Lai WC, Cheng LP (2009). Formation of microporous poly (hydroxybutyric acid) membranes for culture of osteoblast and fibroblast.. Polym Adv Technol..

[23] Thirumala S, Gimble JM, Devireddy RV (2010). Evaluation of methylcellulose and dimethyl sulfoxide as the cryoprotectants in a serum-free freezing media for cryopreservation of adipose-derived adult stem cells.. Stem Cells Dev..

[24] Nemir S, West JL (2010). Synthetic materials in the study of cell response to substrate rigidity.. Ann Biomed Eng..

[25] Zhu L, Wool RP (2006). Nanoclayreinforcedbio-based elastomers: Synthesis and characterization.. Polymer..

[26] Li Z, Zhang J, Chen S (2008). Effects of carbon blacks with various structures on vulcanization and reinforcement of filled ethylene-propylene-diene rubber.. Express Polym Lett..

[27] Razzaghi-Kashani M, Gharavi N, Javadi S (2008). The effect of organo-clay on the dielectric properties of silicone rubber.. Smart Mater Struct..

[28] Wang J, Chen Y (2008). Preparation of an organomontmorillonite master batch and its application to high‐temperature‐vulcanized silicone‐rubber systems.. J Appl Polym Sci..

[29] Lyssiotis CA, Lairson LL, Boitano AE, Wurdak H, Zhu S, Schultz PG (2011). Chemical control of stem cell fate and developmental potential.. Angew Chem Int Ed Engl..

